# Genome sequence and assembly of the amylolytic *Bacillus licheniformis* T5 strain isolated from Kazakhstan soil

**DOI:** 10.1186/s12863-023-01177-8

**Published:** 2024-01-02

**Authors:** Arman Mussakhmetov, Assel Kiribayeva, Asset Daniyarov, Aitbay Bulashev, Ulykbek Kairov, Bekbolat Khassenov

**Affiliations:** 1 National Center for Biotechnology, 13/5 Korgalzhyn Road, Astana, 010000 Kazakhstan; 2 Laboratory of Bioinformatics and Systems Biology, Center for Life Sciences, National Laboratory Astana, Nazarbayev University, 53 Kabanbay Batyr Avenue, Astana, 010000 Kazakhstan; 3 S. Seifullin Kazakh Agrotechnical Research University, 62 Zhenis Avenue, Astana, 010001 Kazakhstan; 4 Faculty of Natural Sciences, L.N. Gumilyev, Eurasian National University, 2 Kanysh Satpayev Street, Astana, 010008 Kazakhstan

**Keywords:** *Bacillus licheniformis* strain, Oxford Nanopore sequencing, Genome assembly, Enzymatic activities, Kazakhstan

## Abstract

**Objectives:**

The data presented in this study were collected with the aim of obtaining the complete genomes of specific strains of *Bacillus* bacteria, namely, *Bacillus licheniformis* T5. This strain was chosen based on its enzymatic activities, particularly amylolytic activity. In this study, nanopore sequencing technology was employed to obtain the genome sequences of this strain. It is important to note that these data represent a focused objective within a larger research context, which involves exploring the biochemical features of promising Bacilli strains and investigating the relationship between enzymatic activity, phenotypic features, and the microorganism's genome.

**Data description:**

In this study, the whole-genome sequence was obtained from one *Bacillus* strain, *Bacillus licheniformis* T5, isolated from soil samples in Kazakhstan. Sample preparation and genomic DNA library construction were performed according to the Ligation sequencing gDNA kit (SQK-LSK109) protocol and NEBNext module. The prepared library was sequenced on a MinION instrument (Oxford Nanopore Technologies nanopore sequencer with a maximum throughput of up to 30 billion nucleotides per run and no limit on read length), using a flow cell for nanopore sequencing FLO-MIN106D. The genome de novo assembly was performed using the long sequencing reads generated by MinION Oxford Nanopore platform. Finally, one circular contig was obtained harboring a length of 4,247,430 bp with 46.16% G + C content and the mean contig 428X coverage. *B. licheniformis T5* genome assembly annotation revealed 5391 protein-coding sequences, 81 tRNAs, 51 repeat regions, 24 rRNAs, 3 virulence factors and 53 antibiotic resistance genes. This sequence encompasses the complete genetic information of the strain, including genes, regulatory elements, and noncoding regions. The data reveal important insights into the genetic characteristics, phenotypic traits, and enzymatic activity of this *Bacillus* strain.

The findings of this study have particular value to researchers interested in microbial biology, biotechnology, and antimicrobial studies. The genomic sequence offers a foundation for understanding the genetic basis of traits such as endospore formation, alkaline tolerance, temperature range for growth, nutrient utilization, and enzymatic activities. These insights can contribute to the development of novel biotechnological applications, such as the production of enzymes for industrial purposes.

Overall, this study provides valuable insights into the genetic characteristics, phenotypic traits, and enzymatic activities of the *Bacillus licheniformis* T5 strain. The acquired genomic sequences contribute to a better understanding of this strain and have implications for various research fields, such as microbiology, biotechnology, and antimicrobial studies.

**Supplementary Information:**

The online version contains supplementary material available at 10.1186/s12863-023-01177-8.

## Objective

The objective of this study was to utilize Oxford Nanopore sequencing technology to obtain the complete genome sequences of a specific *Bacillus* strain, *Bacillus licheniformis* T5, which is known for its amylolytic activity. This strain exhibits significant potential for industrial applications [1]*. Bacillus licheniformis* T5 produces a thermostable α-amylase with high pH stability. The utilization of Oxford Nanopore sequencing allows long read sequencing and rapid acquisition of genomic data, enabling a comprehensive analysis of the genetic factors underlying the enzymatic activities of this strain.

Bacilli species are renowned for their diverse enzymatic capabilities, making them valuable in various industries, such as biocatalysis, hydrolysis of proteins [2], degradation of plant polymers [3], biofuel production, and starch food processing [4]. The amylases derived from this *Bacillus* strain have found extensive industrial applications. *B. licheniformis* T5 with specific enzymatic activity holds immense potential for optimization and utilization in these sectors [5].

The comprehensive analysis of the complete genome of *Bacillus licheniformis* T5 offers valuable insights into the genetic basis of its enzymatic activity. This knowledge can be utilized for genetic engineering approaches and optimization strategies, ultimately enhancing the industrial applications of this strain.

In summary, this study focuses on the application of Oxford Nanopore sequencing technology to obtain the complete genome sequence of *Bacillus licheniformis* T5. This strain possesses amylolytic activity and demonstrates significant potential for industrial use. The genomic information acquired through this study contributes to our understanding of the genetic factors underlying the enzymatic capabilities, facilitating further research and applications in diverse industrial sectors.

## Data description

Genomic sequences obtained from *Bacillus licheniformis* strain T5 were acquired in this study. This strain was isolated from soil samples collected in Kazakhstan. The data revealed that the strain has the ability to form endospores and can thrive within a temperature range of 30–60 °C. It exhibited growth on various nutrient media, including nutrient broth/agar, Luria–Bertani medium, and Mueller–Hinton agar. Additionally, the *B. licheniformis* T5 strain was found to be alkaline-tolerant and capable of growing within a pH range of 5.5–8.0. Furthermore, this strain displayed sensitivity to several antibiotics, including clindamycin, rifampicin, erythromycin, ciprofloxacin, tobramycin, tetracycline, penicillin, gentamicin, ampicillin, kanamycin, streptomycin, and chloramphenicol [6].

The genomic sequences provide a comprehensive representation of the genetic information present in *Bacillus licheniformis* T5. This sequence encompasses the complete set of genes, regulatory elements, and noncoding regions that constitute their genomes.

During a 5-day culture period on Difco sporulation medium (Sigma-Aldrich, UK), Arret-Kirshbaum sporulation agar, and modified nutrient agar, the strain was observed to develop endospores. This strain possesses distinct enzymatic characteristics. *B. licheniformis* T5 displays amylolytic activity, producing thermostable α-amylase when grown on starch medium, with maximum activity observed at 80 °C and pH 6.0. The α-amylase isolated from *B. licheniformis* T5 has a number of features that distinguish it favorably from α-amylases isolated from related strains: α-amylase retains 100% activity after preliminary incubation of the enzyme for 10 h in buffers with pH from 6 to 12; retains 100% activity in the presence of 1% β-mercaptoethanol and is not inhibited by SDS at concentrations of 10 and 20 mM. These indicators allow us to consider α-amylase as a promising enzyme for industrial use, and the *B. licheniformis* T5 strain as a producer strain.

To process the data, the collected strain was cultured in 10 mL of nutrient broth (Himedia, India) in a shaker incubator at 37 °C and 150 rpm for 18 h. Following culture, cells were collected via centrifugation at 6000 × g, and genomic DNA was isolated using a Genomic DNA Purification Kit (Promega, USA) according to the manufacturer's protocol. The quality of the isolated DNA was assessed through spectrophotometry and agarose gel electrophoresis.

**Fig. 1:**
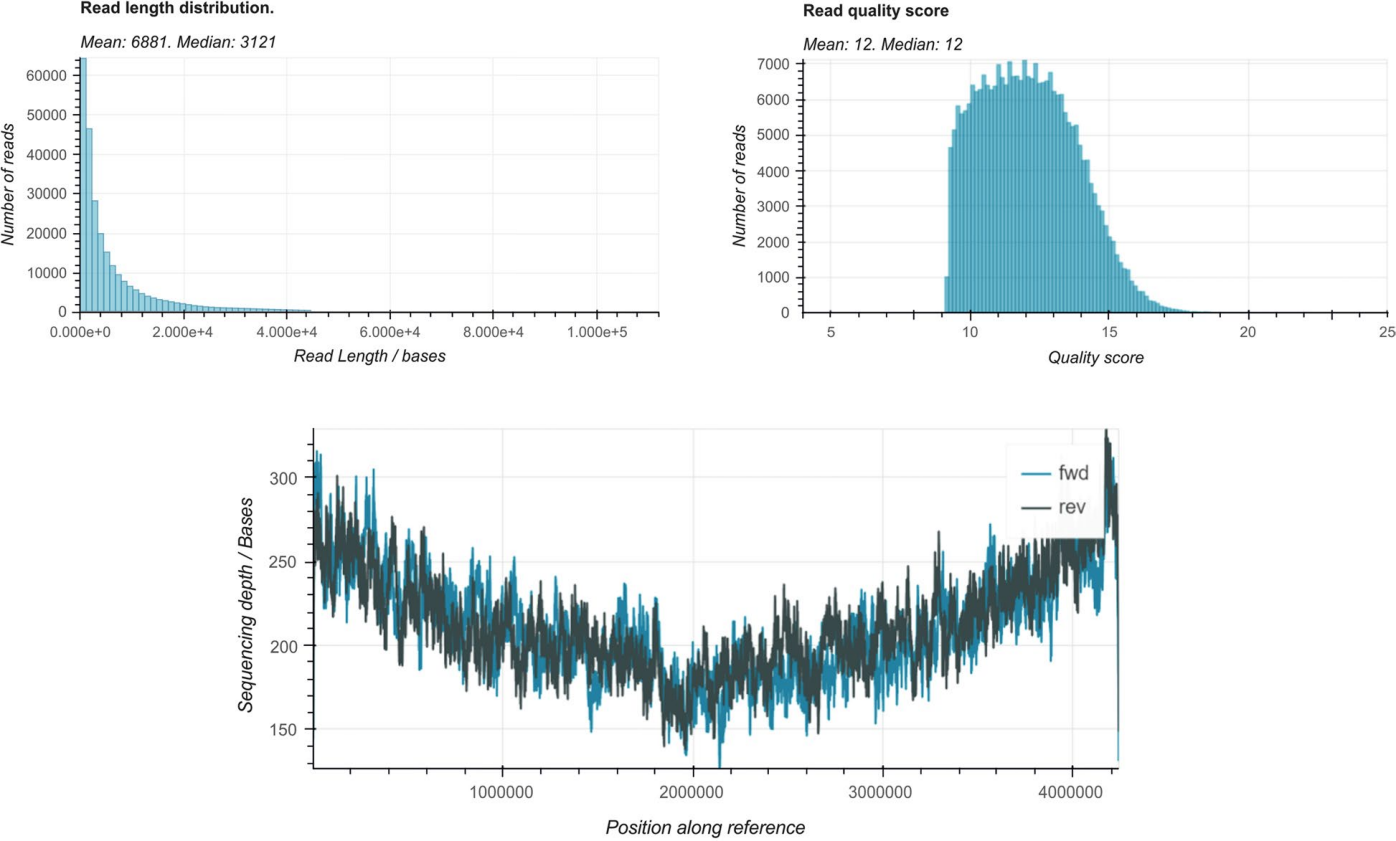
*Bacillus licheniformis T5 strain* ONT sequencing read quality control and genome coverage representation

Construction of genomic DNA libraries was performed using the Oxford Nanopore Technologies (ONT) sequencing kit (SQK-LSK109). This involved DNA fragmentation followed by adapter ligation [7]. The resulting libraries were quantified using Qubit 2.0 (Invitrogen, USA) and subjected to sequencing on a MinION platform (https://nanoporetech.com/) with a FLO-MIN106D flow cell (R9). Raw sequence files were processed using Guppy v3.4.1 to call the reads, and low-quality reads were removed from further analysis. A total of 262,436 reads with a mean read length of 3121 bp and a mean read quality of 12.11 were obtained [8] (Fig. 1). The Epi2me “epi2me-labs/wf-bacterial-genomes” pipeline was implemented to perform further analysis and assembly. The genome de novo assembly was performed using Flye v.2.9.1 [9] designed for the long sequencing reads generated by ONT. One circular contig was obtained harboring a length of 4,247,430 bp with 46.16% G + C content and high mean 428X contig coverage. The resulting sequences from ONT were annotated using Prokka rapid prokaryotic genome annotation (Prokka v1.13.7) [10] and DNA Features Viewer software v.3.1.2. Additional genome annotation and genome circular visualization [8] were performed using the PATRIC [11] which is now readily accessible through the BV-BRC [12] platforms (https://www.bv-brc.org/). The BUSCO v5.4.7 [13] tool was used to assess the final genome assembly. Genome assembly annotation revealed 5391 protein-coding sequences, 81 tRNAs, 51 repeat regions, 24 rRNAs, 3 virulence factors and 53 antibiotic resistance genes (according to PATRIC DB) [14].

The resulting genome assembly of *Bacillus licheniformis* T5 has been deposited in NCBI GenBank with the accession number CP124852 under the Bioproject number PRJNA967102, as shown in Table 1 [15].

**Table 1:** Overview of data files/data sets

Label	Name of data file/data set	File types (file extension)	Data repository and identifier (DOI or accession number)
Data file 1	Genome assembly of *Bacillus licheniformis strain* T5	Fasta file (.fasta)	NCBI GenBank BioProject: https://identifiers.org/ncbi/bioproject:PRJNA967102 [15]
Data file 2	ONT sequencing read quality control, genome coverage and circular genome representation of *Bacillus licheniformis strain* T5	Word file (.docx)	Figshare https://doi.org/10.6084/m9.figshare.24018087 [8]
Data file 3	Read and assembly statistics, Genome statistics, features and specialty genes of *Bacillus licheniformis strain* T5	Word file (.docx)	Figshare https://doi.org/10.6084/m9.figshare.24025071 [14]
Data file 4	Genome assembly annotation results	Excel file (.xlsx)	Figshare https://doi.org/10.6084/m9.figshare.24025113 [16]

**Fig. 2:**
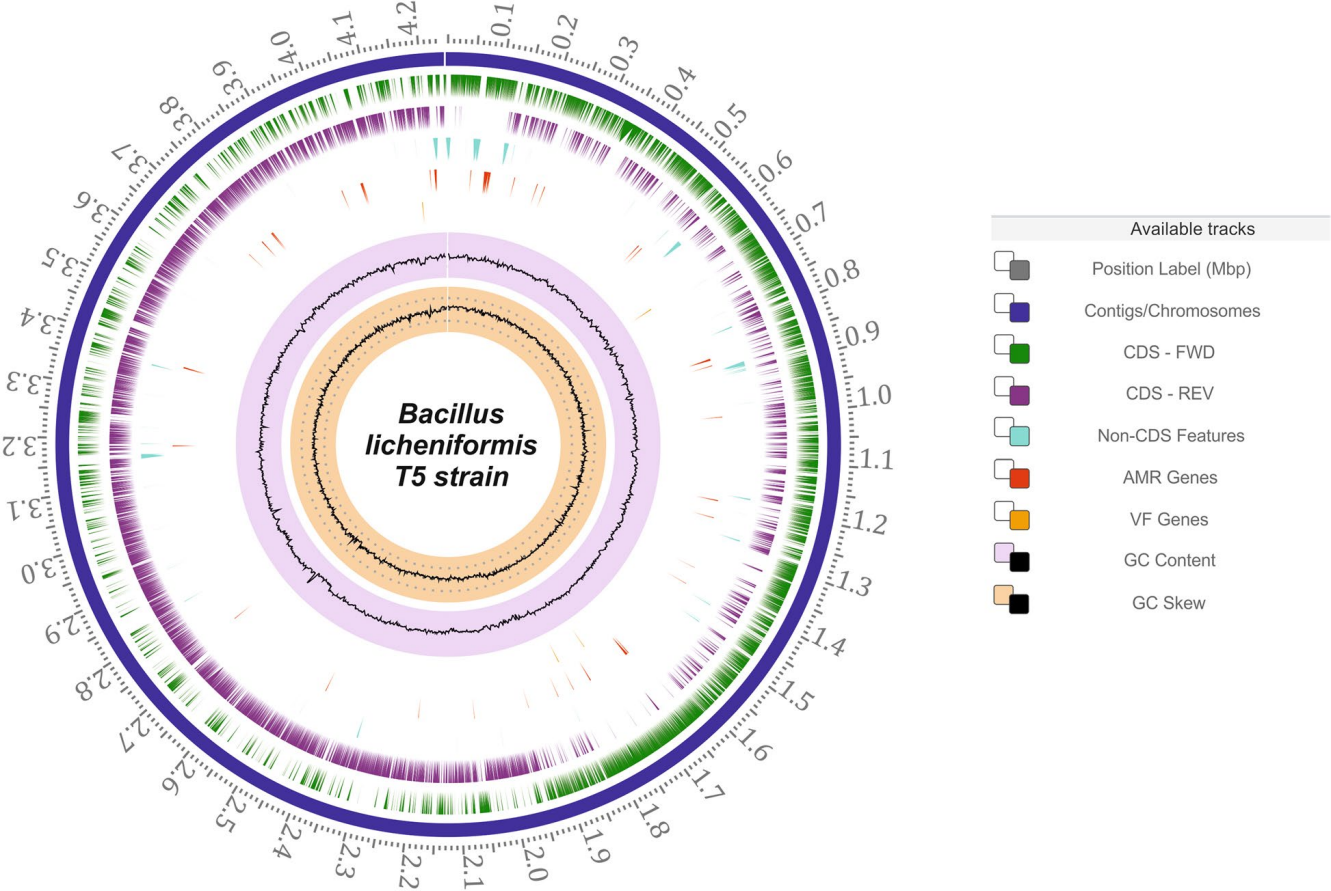
Circular genome representation of *Bacillus licheniformis T5 strain*

In summary, the data collected in this study involve the genome sequence assembly and annotation [16] of *Bacillus licheniformis* T5 (Fig. 2). This assembly offers a comprehensive understanding of the genetic information within this strain, including the presence of specific genetic elements responsible for observed phenotypic traits. The data were processed through various steps, such as bacterial cultivation and growth observation under different conditions, DNA isolation, library preparation, Oxford Nanopore sequencing, base calling, genome de novo assembly and genome annotation. The resulting assembly has been deposited in NCBI GenBank and is freely available for further comparative studies and explorations.

## Limitations

Oxford Nanopore technology has been used to generate the genome sequences of *B. licheniformis* T5 strain in order to ensure complete assembly. Nanopore sequencing differs from these earlier methods in that it directly detects nucleotides without active DNA synthesis, as a long stretch of single-stranded DNA passes through a protein nanopore stabilized by an electrically refractory polymer membrane [17, 18]. The nanopore sequencing does not require imaging apparatus to detect nucleotides, making the system portable and significantly reduces the initial cost of the full-genome sequencing [19]. The important elements of nanopore sequencing are a membrane with nanometer-sized pores and a chamber filled with an electrolytic solution. The principle of operation is that when nucleotides pass through the pore, the cross-section available for ions decreases and the current strength, which is measured, falls accordingly [20]. The accuracy of the method is determined by the number of times the DNA chain passes through the pore [21]. Genome assembly, analysis and further annotation were performed using novel, robust and validated bioinformatics methods and tools. Whole-genome was sequenced with high coverage and the final genome assembly finalized as the one circular contig [14]. Therefore, the authors are not aware of any limitations in the data.

### Supplementary Information

**Additional file 1:** **Table S1.** Genome statistics of *Bacillus licheniformis T5 strain. ***Table S2.** Genomic features of*Bacillus licheniformis T5 strain. ***Table S3.** Specialty genes of *Bacillus licheniformis T5 strain. ***Table S4. **Read and assembly statistics of *Bacillus licheniformis T5 strain.*

**Additional file 2.** 

## Data Availability

The data described in this manuscript are available and openly accessed on NCBI GenBank under Bioproject no. PRJNA967102. Please see Table 1 and references [8, 14–16] for details and links to the data.

## Abbreviations

ONT: Oxford Nanopore Technology
DNA: Deoxyribonucleic acid
bp: Base pair
NCBI: National Center for Biotechnology Information
