# Supplementary material for: Genome sequence and assembly of the amylolytic Bacillus licheniformis T5 strain isolated from Kazakhstan soil
Source: BMC Genom Data. 2024 Jan 2;25:3. doi: 10.1186/s12863-023-01177-8 (PMC10759562; doi:10.1186/s12863-023-01177-8)
Supplement: Supplementary file 2 — Additional file 2. [file 12863_2023_1177_MOESM2_ESM.docx]

**Supplementary material S1**

**Table S1. Genome statistics of *Bacillus licheniformis T5 strain***

| **Genome Statistics** | |
| --- | --- |
| Contigs | [1](https://www.bv-brc.org/view/Genome/1386.2682#view_tab=sequences) |
| Genome Length | 4247430 |
| GC Content | 46.157322 |
| Contig L50 | 1 |
| Contig N50 | 4247430 |
| **Genome Quality** | |
| Coarse Consistency | 99.6 |
| CheckM Completeness | 99.7 |
| Genome Quality | Good |

**Table S2. Genomic features of *Bacillus licheniformis T5 strain***

| Type | BV-BRC |
| --- | --- |
| CDS | [5391](https://www.bv-brc.org/view/Genome/1386.2682#view_tab=features&filter=and(eq(feature_type,CDS),eq(annotation,PATRIC))) |
| tRNA | [81](https://www.bv-brc.org/view/Genome/1386.2682#view_tab=features&filter=and(eq(feature_type,tRNA),eq(annotation,PATRIC))) |
| repeat_region | [51](https://www.bv-brc.org/view/Genome/1386.2682#view_tab=features&filter=and(eq(feature_type,repeat_region),eq(annotation,PATRIC))) |
| rRNA | [24](https://www.bv-brc.org/view/Genome/1386.2682#view_tab=features&filter=and(eq(feature_type,rRNA),eq(annotation,PATRIC))) |

**Table S3. Specialty genes of *Bacillus licheniformis T5 strain***

| Type | Source | Genes |
| --- | --- | --- |
| Virulence Factor | PATRIC_VF | [3](https://www.bv-brc.org/view/Genome/1386.2682#view_tab=specialtyGenes&filter=and(eq(property,%22Virulence%20Factor%22),eq(source,%22PATRIC_VF%22))) |
| Antibiotic Resistance | PATRIC | [53](https://www.bv-brc.org/view/Genome/1386.2682#view_tab=specialtyGenes&filter=and(eq(property,%22Antibiotic%20Resistance%22),eq(source,%22PATRIC%22))) |

**Table S4. Read and assembly statistics of *Bacillus licheniformis T5 strain***

|  | Median read length (bp) | Mean read quality | Read data (Mb) | # contigs | Largest contig (Mb) | Total length (Mb) | Reference length (Mb) | Mean contig coverage | # circular contigs |
| --- | --- | --- | --- | --- | --- | --- | --- | --- | --- |
| Bacillus licheniformis T5 | 3121.0 | 12.110542 | 1805.94 | 1 | 4.25 | 4.25 | 4.27 | 428.0 | 1 |
